# Recording long-term potentiation of synaptic transmission by three-dimensional multi-electrode arrays

**DOI:** 10.1186/1471-2202-7-61

**Published:** 2006-08-30

**Authors:** Maksym V Kopanitsa, Nurudeen O Afinowi, Seth GN Grant

**Affiliations:** 1 Genes to Cognition Programme, The Wellcome Trust Sanger Institute, Genome Campus, Hinxton, Cambridge CB10 1SA, UK

## Abstract

**Background:**

Multi-electrode arrays (MEAs) have become popular tools for recording spontaneous and evoked electrical activity of excitable tissues. The majority of previous studies of synaptic transmission in brain slices employed MEAs with planar electrodes that had limited ability to detect signals coming from deeper, healthier layers of the slice. To overcome this limitation, we used three-dimensional (3D) MEAs with tip-shaped electrodes to probe plasticity of field excitatory synaptic potentials (fEPSPs) in the CA1 area of hippocampal slices of 129S5/SvEvBrd and C57BL/6J-Tyr^C-Brd ^mice.

**Results:**

Using 3D MEAs, we were able to record larger fEPSPs compared to signals measured by planar MEAs. Several stimulation protocols were used to induce long-term potentiation (LTP) of synaptic responses in the CA1 area recorded following excitation of Schäffer collateral/commissural fibres. Either two trains of high frequency tetanic stimulation or three trains of theta-burst stimulation caused a persistent, pathway specific enhancement of fEPSPs that remained significantly elevated for at least 60 min. A third LTP induction protocol that comprised 150 pulses delivered at 5 Hz, evoked moderate LTP if excitation strength was increased to 1.5× of the baseline stimulus. In all cases, we observed a clear spatial plasticity gradient with maximum LTP levels detected in proximal apical dendrites of pyramidal neurones. No significant differences in the manifestation of LTP were observed between 129S5/SvEvBrd and C57BL/6J-Tyr^C-Brd ^mice with the three protocols used. All forms of plasticity were sensitive to inhibition of *N*-methyl-*D*-aspartate (NMDA) receptors.

**Conclusion:**

Principal features of LTP (magnitude, pathway specificity, NMDA receptor dependence) recorded in the hippocampal slices using MEAs were very similar to those seen in conventional glass electrode experiments. Advantages of using MEAs are the ability to record from different regions of the slice and the ease of conducting several experiments on a multiplexed platform which could be useful for efficient screening of novel transgenic mice.

## Background

The dynamically changing strength of connections between neurones was proposed to be a mechanism for memory formation more than a century ago [1-3]. In 1949, Hebb provided a theoretical framework for this hypothesis [4] and in the 1960s this concept gained crucial experimental support when it was discovered that neurones can alter their firing properties upon experiencing particular patterns of external stimulation, i.e. they exhibit synaptic plasticity [5]. A classical example of synaptic plasticity is long-term potentiation (LTP) discovered in the dentate gyrus more than three decades ago [6]. Traditionally, LTP is defined as a prolonged enhancement of synaptic responses of a neurone or a neuronal ensemble after short periods of high-frequency stimulation [7]. Properties of LTP such as permanence, associativity and input specificity suggested that it could be a physiological basis of certain types of memory [8-11]. The straightforward relationship between LTP and memory has recently been questioned [12], however in the amygdala and hippocampus, learning has been found to involve increases of the synaptic output [13,14]. As LTP and cognitive functions often rely on common membrane ion channels and signalling pathways [15-17], *in vitro *studies of synaptic plasticity are vital for characterisation of mutant animals with potential cognitive disturbances [18,19]. Mutant mice have become a standard tool for dissection of the molecular basis of LTP especially since pharmacological reagents are available to only a subset of synapse proteins. The Genes to Cognition programme aims to systematically study LTP in 100 mutant mouse lines where components of synaptic complexes are disrupted [20]. With large scale mouse mutagenesis programs now in progress [21], some with the aim of disrupting all genes in the mouse genome (European Union Conditional Mouse Mutant Program) [22], it is essential to have rapid and scalable tools for studying LTP in hundreds (or perhaps thousands) of mutant mouse lines.

Another problem is the absence of standard experimental procedures in the LTP field. Most common LTP experiments performed in the synaptic pathway between CA3 and CA1 pyramidal neurones of the hippocampus rely on a multitude of induction protocols (A. Howell, M. Marshall, S. Grant, manuscript in preparation). Moreover, information about the position of stimulating and recording electrodes with respect to each other and in relation to the laminar structure of the hippocampus is lacking in many reports making their cross-comparison difficult.

Such problems can be at least partially overcome when multi-electrode arrays (MEAs) are employed [23]. They have become popular tools for recording spontaneous and evoked activity from excitable tissues [24-28]. For the purposes of LTP analysis, an orderly arrangement of MEA electrodes allows for a precise geometrical assignment of stimulation and recording sites. Current commercial hardware permits the operation of several MEA set-ups by a single computer effectively increasing the output and minimising the number of animals needed for long-term LTP experiments. Several reports have demonstrated the possibility of inducing LTP in hippocampal slices using MEAs [29-34]. However, no systematic study of different induction protocols using MEA technology has yet been published. Furthermore, in all these studies, planar MEAs were employed that could not record activity from the deeper, presumably healthier layers of the slice. In this study, we present our data on the LTP of extracellular field potentials recorded by MEAs with three-dimensional tip-shaped electrodes which allow the recording of larger signals and injection of stronger currents in slice tissue [35]. We show that there is a gradient of the degree of LTP in stratum radiatum (SR) of the CA1 area of hippocampal slices of 129S5/SvEvBrd (129S5) and albino C57BL/6J-*Tyr*^C-Brd ^(C57) mice. These strains were chosen as they are mainstays of large scale mouse knockout programmes [36]. In this work, we test three different LTP-inducing protocols and investigate sensitivity of LTP to the inhibition of *N*-methyl-D-aspartate (NMDA) receptors.

## Results

### Properties of field excitatory postsynaptic potentials in CA1 area of the hippocampus recorded by 3D MEAs

**Figure 1:**
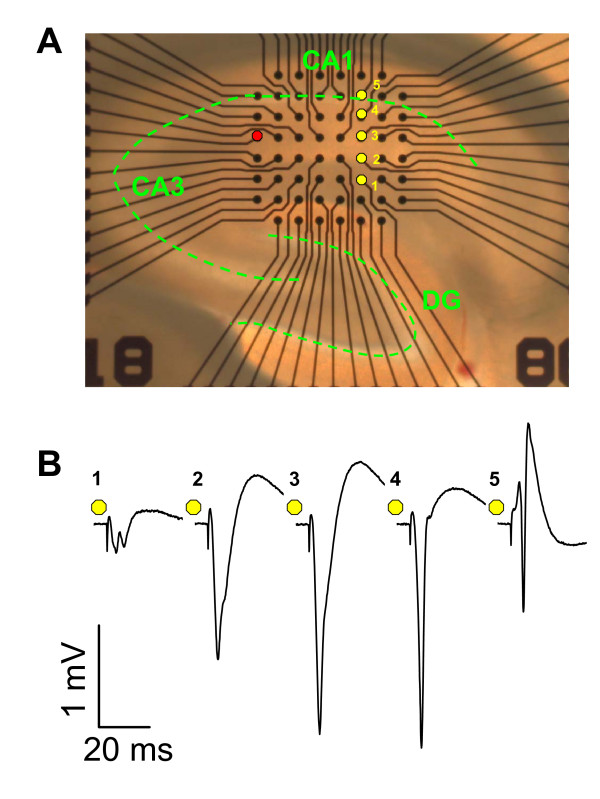
**Laminar profile of fEPSPs in CA1 area of the hippocampus recorded by a multi-electrode array**. **A) **A 350 μm thick hippocampal slice was placed over 8 × 8 3D MEA and a suitable electrode (red circle) was selected for stimulation of Schäffer collateral/commissural fibres. A column of electrodes (yellow circles) aligned in parallel with the direction of apical dendrites of CA1 pyramidal neurones was chosen to record synaptic responses in *stratum lacunosum-moleculare *(electrode 1), *stratum radiatum *(electrodes 2–4) and *stratum pyramidale/stratum oriens *(electrode 5). Layers of cell bodies are delineated by green dashed line. Distance between electrodes was 100 μm. **B) **Waveforms of fEPSPs recorded by electrodes 1–5 in response to a voltage step (biphasic positive/negative 3.5 V pulse, 100 μs/phase) applied to stimulation electrode. Initial small, negative deflections are the stimulus artefacts.

To record evoked field excitatory postsynaptic potentials (fEPSPs), we placed 350 μM thick brain slices containing the hippocampal region over an 8 × 8 3D MEA with an inter-electrode distance of 100 μm (Fig. 1A). Monopolar stimulation of Schäffer collateral/commissural fibres was performed through an electrode located on the border between areas CA1 and CA3. Biphasic positive-negative (100 μs/phase) voltage steps up to 3.7 V applied to the stimulation electrode evoked fEPSPs that could be recorded across the entire CA1 area. Fig. 1B demonstrates a characteristic laminar profile of fEPSPs recorded by a column of electrodes aligned perpendicular to *stratum pyramidale *(SP). Large negative deflections of the extracellular potential in SR represent synaptic activation of apical dendrites of pyramidal cells (electrodes 2–4 in Fig. 1). Responses in *stratum lacunosum-moleculare *(SLM) were usually smaller (electrode 1, Fig. 1). In contrast to apical dendritic zones, the initial membrane response showed positive polarity in SP and above the layer of pyramidal cell bodies (Fig. 1B, electrode 5). At maximum stimulation strengths, a fast negative deflection, a population spike [37], was often seen overriding the slower positive wave (Fig. 1B, electrode 5). We did not observe population spikes consistently in all slices probably due to the limited maximum voltage step (<3.7 V) that could be applied thorough MEA electrodes (see Additional File 1). Stronger stimulation led to large Faradaic effects on electrodes causing artefacts. A distinctive feature of MEA recordings was that negative fEPSPs did not just return to the baseline, but were followed by a slow positive wave (Fig. 1B). Such positivities are usually attributed to closeness of a recording electrode to the pyramidal cell layer (e.g. [14]), however in our case late positive waves were consistently observed in deeper parts of SR (Fig. 1B). Moreover, a reverse fEPSP waveform, positive and then negative, was usually recorded in *stratum oriens *(SO), suggesting that this secondary wave was a passive consequence of the synaptic activation. Similar positive shoulders have been observed in other studies that employed MEAs (e.g. see Fig. 4B in [31] or Fig. 1A in [38]).

**Figure 2:**
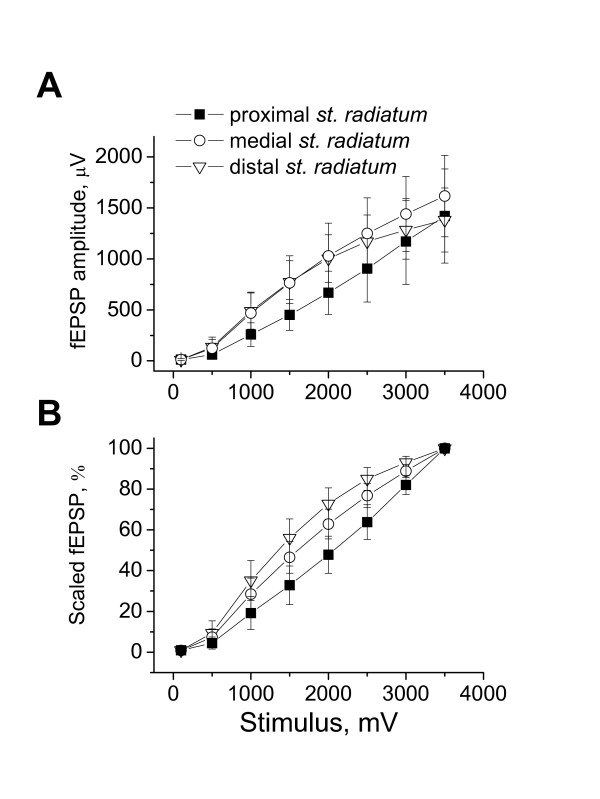
**Input/output relationships recorded in proximal, medial and distal parts of SR**. **A) **Relationships between stimulus strength and fEPSP amplitudes in different locations within SR were determined in 19 hippocampal slices from ten 129S5 mice. Data on this and other Figures are presented as mean ± standard deviation. **B) **Same data as in (A), but normalised by fEPSP amplitude evoked by maximum 3.5 V stimulus.

In the search for possible regional differences between fEPSPs, we analysed input-output (I/O) relationships for 19 hippocampal slices obtained from ten 129S5 mice. Slices were placed on MEA100 3D arrays so that there were at least three recording electrodes in SR, with the most proximal one being within 50–80 μm from SP. Two other electrodes, designated as medial and distal, were therefore 100 and 200 μm further down from a proximal one along a line perpendicular to SP. I/O curves demonstrate that true saturation of the synaptic strength could not be obtained for any location (Fig. 2A). The steepest rise of the I/O relationship was noted for distal fEPSPs (Fig. 2B). The recording location interacted with the stimulus strength (*F*_(14,252) _= 8.15; *P *< 0.0001). Bonferroni/Dunn's *post hoc *tests revealed that proximal fEPSPs were significantly smaller (*P *< 0.01) than responses recorded in either medial or distal locations for stimulus strengths between 0.5 to 2.5 V (Fig. 2A). At 3 V, proximal fEPSPs differed only from the medial fEPSPs (*P *< 0.05) and at 3.5 V there were no differences between recording locations.

Similar results were obtained for I/O relationships in 13 slices from C57 mice (data not shown).

### Long-term potentiation of synaptic responses in SR varies with recording location

**Figure 3:**
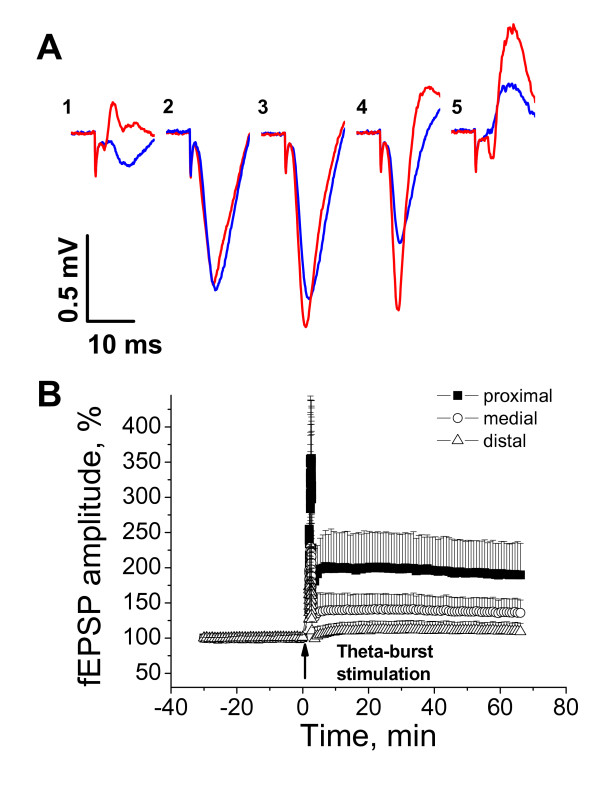
**TBS-induced LTP in hippocampal slices from 129S5 mice recorded by 8 × 8 3D MEAs**. A) Exemplar records of fEPSPs were obtained shortly before and 60 min after induction of LTP (blue and red traces respectively) from five CA1 locations spatially similar to those, outlined in Fig. 1A. LTP was induced by three trains of TBS with intra-train interval of 20 s. There were four pulses at 100 Hz within a burst and 10 bursts delivered at 5 Hz per train. Stimulus strength originally set at evoking 40% of the maximal proximal fEPSP was increased to 125% during TBS. Note a pronounced potentiation of proximal fEPSP (electrode 4) in the absence of fully blown population spike in SP/SO. Slight potentiation of fEPSP was observed in the middle of SR (electrode 3) and no potentiation was seen in distal apical dendrites (electrode 2). In SLM, fEPSPs were small, so indistinctive changes were seen upon LTP induction (electrode 1). Photo of the slice from this experiment as well as waveforms of its responses to maximal stimulation can be found in the Additional file 1. B) Summary LTP data obtained while recording simultaneously in three locations in SR in 13 slices from 129S5 mice. Only the upper part of the S.D. error bar is shown for clarity.

**Figure 4:**
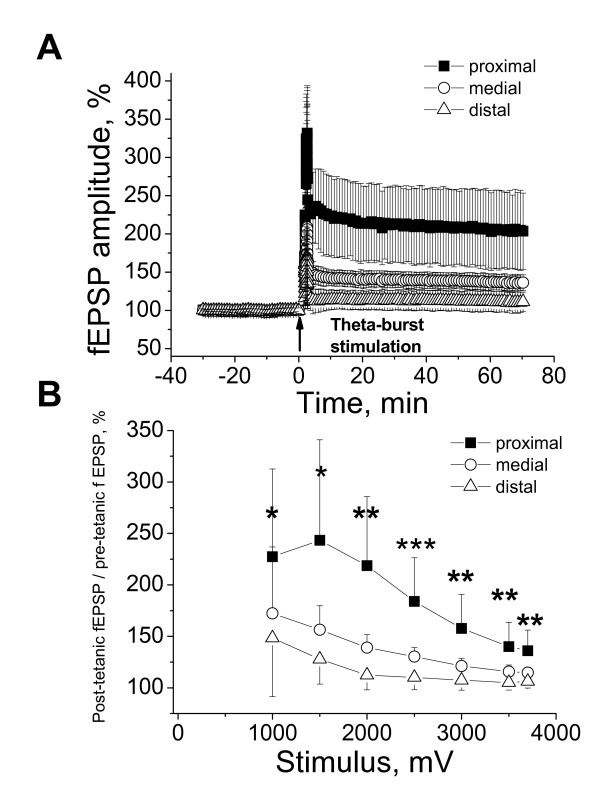
**TBS-induced LTP in hippocampal slices from C57 mice recorded by 8 × 8 3D MEAs**. A) Summary LTP data obtained in simultaneous recordings from proximal, medial and distal locations in *stratum radiatum *in 11 slices from C57 mice. B) Comparative changes of fEPSPs recorded from three different locations in SR before and 60 min after induction of LTP by three trains of TBS. Ratios of post- to pre-TBS fEPSP amplitudes are plotted against corresponding stimulus strengths. Data from two lowest stimulation strengths, 100 and 500 mV are omitted as the often produced zero or a very small response (< 100 μV). Note that the post/pre-LTP ratio is always larger in proximal compared to medial region of SR (Bonferroni/Dunn's post hoc test: * – *P *< 0.01; ** – *P *< 0.001; *** – *P *< 0.0001). Only a half of the S.D. error bar is shown for clarity.

We used a theta-burst stimulation protocol (TBS) to induce LTP of fEPSPs in SR of the CA1 area [39]. Baseline stimulation was set to evoke fEPSPs at about 40% of the maximum response attained in the proximal region of SR. During TBS, stimulus strength was increased to 125% of the baseline value. As shown in Figures 3 and 4, amplitudes of fEPSP remained enhanced 60 min after three trains of TBS (*F*_(1,22) _= 127.89; *P *< 0.0001). Recording location had a significant effect on the potentiation (*F*_(2,44) _= 63.23; *P *< 0.0001), whereas strain did not interact with the recording location (*F*_(2,44) _< 1; *not significant*). In slices from both strains, fEPSPs recorded by proximal electrodes, demonstrated the largest potentiation compared to pre-TBS value (129S5: 190 ± 45 %; *t*_(12) _= -7.16; *P *< 0.001 and C57: 205 ± 51%; *t*_(10) _= -6.75; *P *< 0.0001). Medial fEPSPs increased to a lesser extent, but remained above pre-TBS level (129S5: 136 ± 19 %; *t*_(12) _= -6.33; *P *< 0.001 and C57: 137 ± 10%; *t*_(10) _= -11.05; *P *< 0.0001). Minimal changes were seen at the level of distal electrodes (129S5: 110 ± 12 %; *t*_(12) _= -2.74; *P *< 0.05 and C57: 111 ± 13%; *t*_(10) _= -2.60; *P *< 0.05). Larger potentiation of the proximal fEPSPs could be accounted for by increased propensity to generate population spikes following TBS. However, as illustrated in Fig. 3A, a significant increase of the proximal fEPSP (electrode 4) was not always concomitant with major enhancement of the population spike (electrode 5) which would be capable of affecting apical dendritic regions. In fact, the maximal stimulation of the slice, used for the experiment depicted in Fig. 3A, failed to evoke a population spike before TBS (see Additional file 1). It must be noted however, that submaximal stimulation evoked proportionally bigger responses in the middle of SR (Fig. 2). This could be another reason for the difference in fEPSP potentiation between proximal and medial electrodes. In particular, stimulation that elicited 40% of the maximum proximal response evoked an fEPSP that was approximately 60% of the maximum response attained in the middle of SR (Fig. 2B). Consequently, medial fEPSPs could simply have less potential to increase before they hit a potentiation ceiling. It must be emphasised that percentages are given with respect to maximum attained and not to maximum possible response. The limited amplitude of the largest possible stimulation voltage (3.5 – 3.7 V) and the shape of I/O relationship (Fig. 2) suggest that chosen stimulation strengths actually elicited smaller fractional responses than indicated. Nonetheless, we conducted a series of LTP experiments with lower baseline stimulation strength that evoked 40% of the maximum medial fEPSP response in six slices from 129S5 mice. In this case, potentiation of medial fEPSPs was very small (111 ± 8%) and still much less than that recorded at proximal sites (148 ± 36%; *t*_(5) _= 2.76; *P *< 0.05). Also, by comparing I/O relationships recorded before and 60 min after TBS in C57 mice (Fig. 4B), we noted that relative potentiation of the proximal fEPSPs was greater than that of medial fEPSPs (Fig. 4B) at each voltage pulse in the range of 1 to 3.7 V (*P *< 0.01, Bonferroni/Dunn's test). These observations led us to conclude that fEPSPs recorded in the region of proximal apical CA1 dendrites exhibit the maximal amount of potentiation. Therefore, in the description of the next set of experiments, we limited our attention only to recordings from proximal electrodes.

### Monitoring long-term potentiation in two-pathway stimulation experiments

**Figure 5:**
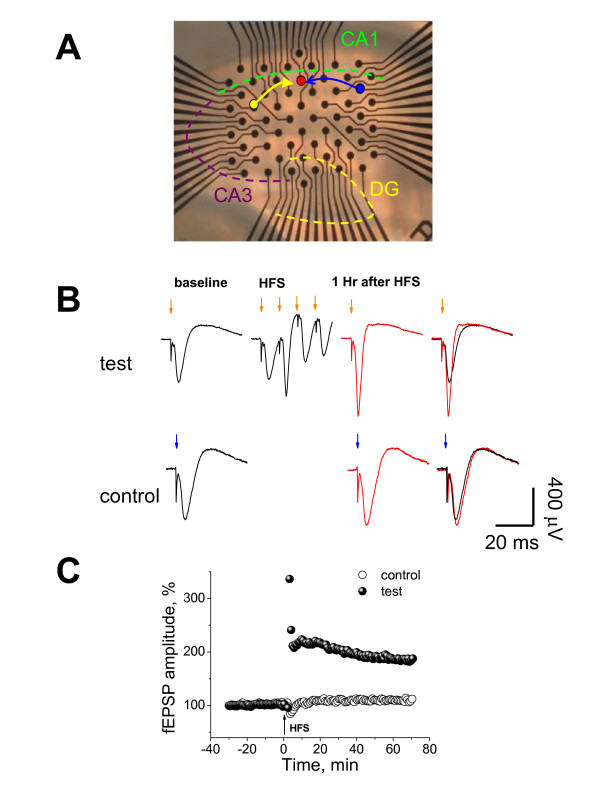
**Scheme of a two-pathway LTP experiment performedusing MEA 5 × 13 3D MEAs**. A) A hippocampal slice was placed over a 5 × 13 3D MEA and a suitable principal recording electrode (red circle) was selected in the proximal part of *stratum radiatum*. Control (blue circle) and test (yellow circle) stimulation electrodes were then chosen on the basis of their pathway independence and distance to the principal recording electrode (usually ≥ 400 μm). Layers of cell bodies in the hippocampal slice are delineated by dashed lines. Distance between electrodes is 140 and 200 μm. B) Alternating stimulation of control and test pathways was performed at baseline stimulus strength that elicited 40% of the maximum fEPSP (first column of traces). After 30 min of stable baseline recording, HFS of the test pathway was performed by two trains of 100 stimuli given at 100 Hz each with inter-train interval of 50 s (trace in the second column). Third column depicts fEPSP recordings 60 min after LTP induction. As follows from overlaid baseline (black) and post-LTP (red) traces in the fourth column, fEPSP amplitude in the test pathway was enhanced, whereas control fEPSPs did not change. C) Plot of fractional fEPSP amplitudes in the control and test pathways before and after LTP induction.

In cases when only one stimulating electrode is used for evoking responses in slices, it is impossible to detect a non-specific drift of baseline conditions if it happens after tetanus. This could potentially lead to inaccurate estimates of LTP. Therefore, in many classical LTP studies, a two-pathway stimulation scheme is used, where a second electrode stimulates a set of fibres independent from the tetanised pathway in order to monitor stability of the slice preparation and to ensure specificity of the induced plasticity [40,41]. To adapt two-pathway stimulation protocols for MEA experiments, we used 5 × 13 3D MEAs with inter-electrode distances of 140 and 200 μm (Fig. 5A). With such an arrangement, electrodes are spread over the entire CA1, thus it becomes easier to select electrodes that are sufficiently apart from each other to activate independent synaptic pathways to CA1 pyramidal neurones. Compared with electrodes in 8 × 8 3D MEA chips, individual electrodes in 5 × 13 3D MEAs are slightly higher (35–45 μm vs. 25–35 μm) and have lower impedance (350–500 kΩ vs. 600–900 kΩ). It was therefore possible to apply slightly stronger voltage (up to 4.5 V) and record larger synaptic potentials. An example of a two-pathway LTP experiment perfomed using an MEA is demonstrated in Fig. 5. Two electrodes, one on the subicular side and another on the CA3 side of the recording location in CA1 were chosen to activate control and test pathways respectively (Fig. 5A). Following 30 min of stable baseline responses in both pathways, tetanic high frequency stimulation (HFS) was delivered through the test electrode (Fig. 5B). HFS consisted of two trains of 100 pulses given at a rate of 100 Hz with 50 s between trains. One hour after HFS, fEPSP amplitude in the test pathway was much larger than before tetanus, whereas no significant change of fEPSP amplitude occurred in the control pathway (Fig. 5B,C). In some recordings we have observed small changes in the amplitude of control fEPSPs after tetanus. Therefore, in all cases we expressed LTP data in a normalised form, dividing the fEPSP amplitude in the tetanised pathway by the amplitude of the control fEPSP for each time point.

**Figure 6:**
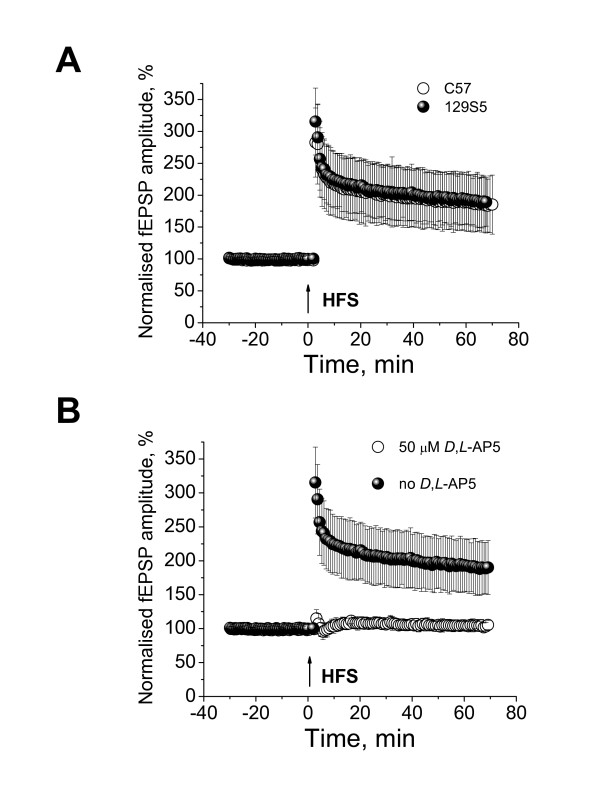
**HFS-induced LTP in hippocampal slices recorded by 5 × 13 3D MEAs**. A) Identical levels of LTP evoked by HFS in slicesfrom 129S5 (*n *= 12) and C57 mice (*n *= 14). B) HFS-induced LTP was inhibited by blocking NMDA receptors. In 5 slices from 129S5 mice, HFS was carried out in the presence of 50 μM *D,L*-AP5, a competitive NMDA receptor antagonist. *D,L*-AP5-treated slices demonstrated a distinctive lack of post-tetanic potentiation and significant reduction of LTP compared to untreated slices (105 ± 7% vs. 191 ± 39 %; *t*_(15) _= -4.79; *P *< 0.001).

As demonstrated in Fig. 6A, HFS led to pronounced LTP of normalised proximal fEPSPs in both 129S5 and C57 strains (129S5: 191 ± 39 %; *t*_(11) _= -8.41; *P *< 0.001 and C57: 187 ± 44%; *t*_(13) _= -7.93; *P *< 0.001). There was no statistical difference in the average amount of potentiation between these strains. In the presence of 50 μM *D*,*L*-2-amino-5-phosphonopentanoic acid (*D*,*L*-AP5), a competitive antagonist of NMDA receptors, HFS failed to induce post-tetanic potentiation and long-term enhancement of fEPSPs (Fig. 6B).

**Figure 7:**
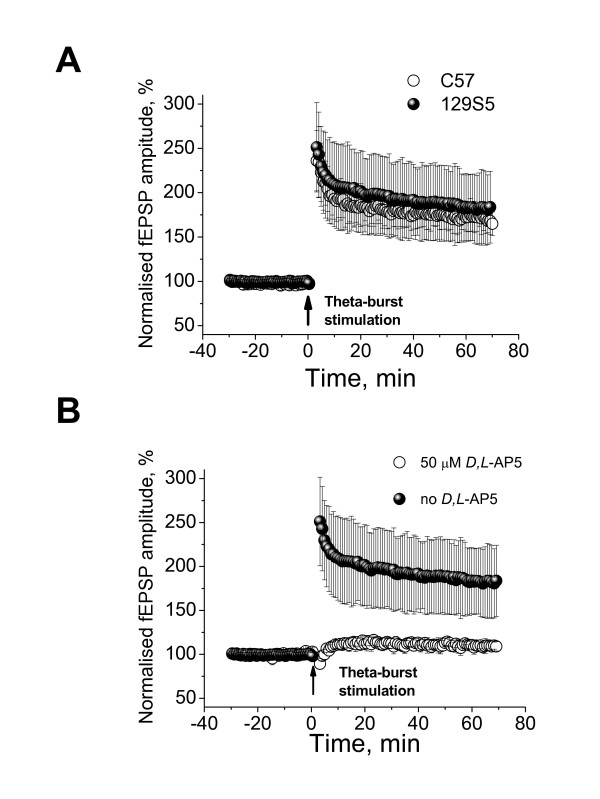
**Theta burst-induced LTP in hippocampal slicesrecorded by 5 × 13 3D MEAs**. A) Summary LTP data obtained in slices from 129S5 (*n *= 14) and C57 (*n *= 7) mice. Exploiting two-pathway paradigm, we corroborated data from one-pathway experiments (Figs. 3B,4A) on robust LTP induced by TBS. No up-regulation of the stimulus strength during TBS was done when using 5 × 13 3D MEAs. B) Inhibition of NMDA receptors by 50 μM *D,L*-AP5 led to a significant attenuation of TBS-induced LTP in 5 slices from 129S5 mice. Average amplitude of normalised fEPSP 60 min after TBS was significantly less in *D,L*-AP5-treated slices compared to untreated 129S5 group (109 ± 5% vs. 178 ± 35 %; *t*_(17) _= -4.24; *P *< 0.001).

Confirming the results obtained with 8 × 8 3D MEAs (Fig. 3B, 4A), TBS induced notable LTP in recordings performed by 5 × 13 3D MEAs (Fig. 7A; 129S5: 182 ± 38 %; *t*_(14) _= -8.33; *P *< 0.001 and C57: 173 ± 16%; *t*_(6) _= -13.35; *P *< 0.001). It should be noted that while stimulation was raised to 125% of the baseline strength during TBS when using 8 × 8 3D MEAs, we did not additionally increase stimulus strength applying HFS and TBS through electrodes of 5 × 13 3D MEAs. As in the case of HFS, LTP evoked by TBS was nearly fully abolished by blocking NMDA receptors (Fig. 7B).

**Figure 8:**
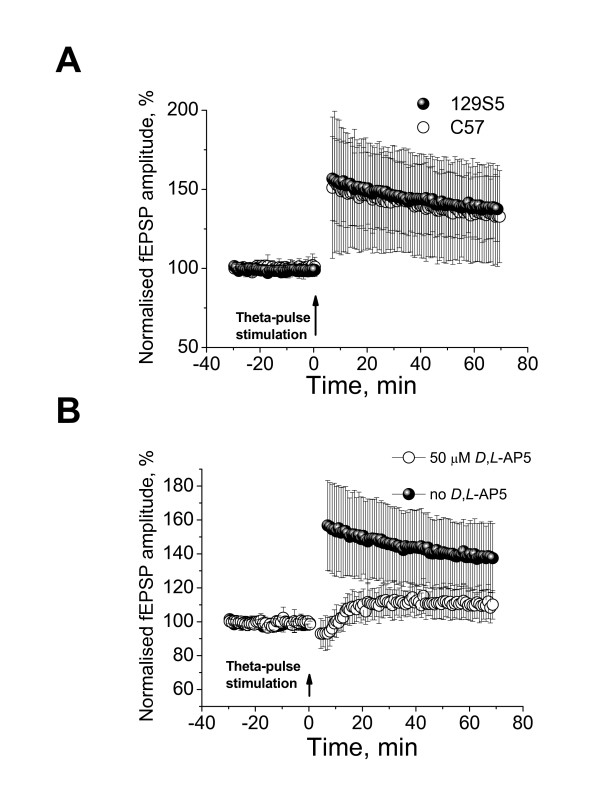
**Theta pulse-induced LTP in hippocampal slicesrecorded by 5 × 13 3D MEAs**. A) LTP was induced by 150 pulses given at 5 Hz in hippocampal slices from 129S5 (*n *= 15) and C57 (*n *= 13) mice. During TPS, amplitude of stimulation was 1.5× of the baseline strength. In the first five minutes following TPS, a transient depression was seen in both control and test pathways (see Additional file 2). Therefore, first five records after TPS were omitted from the plot for clarity. In some cases, TPS did not produce persistent facilitation of fEPSP amplitude that returned to baseline levels after 60 min. However, we pooled data from all slices that exhibited pronounced complex spikes during TPS (see Additional file 2). B) Inhibition of NMDA receptors by 50 μM *D,L*-AP5 led to a significant attenuation of TBS-induced LTP in 5 slices from 129S5 mice. Average amplitude of normalised fEPSP 60 min after TBS was significantly less in *D,L*-AP5-treated slices compared to untreated 129S5 cohort (110 ± 9% vs. 138 ± 20 %; *t*_(19) _= -3.25; *P *< 0.01).

A less widely used protocol to induce LTP is theta-pulse stimulation (TPS) during which afferent fibres are excited at a frequency of 5 Hz for 15–30 s [42,43]. Such stimulation pattern evokes characteristic complex spikes [44] that bear resemblance to spontaneous theta rhythm discharges of CA1 pyramidal neurones observed *in vivo *[45]. In our experimental setting, 30 s of 5 Hz stimulation at baseline strength did not evoke stable LTP probably due to insufficient drive to elicit reliable complex spiking (data not shown). Nonetheless, when we increased the amplitude of stimulation to 150% of baseline strength during TPS, we did observe consistent complex spikes (see Additional file 2) and persistent potentiation of fEPSPs (Fig. 8A: 129S5: 138 ± 20 %; *t*_(14) _= -7.43; *P *< 0.001 and C57: 134 ± 31%; *t*_(12) _= -3.46; *P *< 0.01). In conditions when NMDA receptors were inhibited with *D,L*-AP5, TPS produced only marginal changes to normalised fEPSPs (Fig. 8B: 110 ± 9% in 60 min after LTP induction) confirming this previously reported sensitivity [43,44,46].

## Discussion

In our study, we sought to assess the applicability of 3D MEAs for studies of LTP in mouse hippocampal slices. Previous attempts to use MEA platforms for recording synaptic activity employed planar electrodes that sample signals mostly from the surface of the slice [29-33,47]. In contrast, conventional glass or metal electrode recordings are performed usually at some depth inside the slice tissue where neurones are presumed to be in more physiological, non-damaged conditions. Therefore, we were particularly interested in conducting LTP experiments using 3D, tip-shaped arrays that combine benefits of MEA technology with the possibility to record closer to the source of the signal coming from healthier layers of the slice [35].

Basic features of fEPSPs recorded by means of 3D MEAs were generally in agreement with previous reports on laminar profiles of synaptic responses to excitation of Schäffer collateral/commissural fibres in the CA1 area of the hippocampus *in vitro *[48-50] and *in vivo *[51-53]. Large negative extracellular responses were recorded in SR that reflected synaptic activation of apical dendrites of CA1 pyramidal cells (electrodes 2–4 in Figs 1B, 3A). Pre-synaptic fibre volleys were hard to distinguish and usually more clearly observed following stimulation of the subicular side of CA1 in two-pathway experiments (data not shown). At submaximal stimuli (<3.0 V), fEPSP amplitude was larger in the medial and distal parts of SR (Fig. 2A), possibly pointing to the site of termination of the majority of afferent fibres. At higher strengths of stimulation, there was no statistical difference between sizes of fEPSPs recorded in the three locations within SR. The reason for the latter could be generation of dendritic spikes at proximal apical dendrites that led to disproportionably greater increase of fEPSPs in this location [51,52,54,55]. Weak to moderate stimulation of Schäffer collateral/commissural fibres caused positive deflections of the extracellular fields in SP and SO (electrode 5 in Figs 1B, 3A) indicating intracellular spread of synaptic current into the opposing dendritic arborisation [48]. At larger stimuli, a population spike [37] was observed in many but not all slices (e.g. electrode 5 in Figs 1B; .Additional file 2).

In contrast to many reports that used planar MEAs for fEPSP measurements, application of 3D MEAs permitted routine recording of synaptic potentials of several millivolts in amplitude (Figs. 1B; Additional file 2), which to the best of our knowledge were among the largest ever reported in MEA experiments. Effective excitation of nerve fibres through tip-shaped electrodes of 3D MEAs allowed us to select recording electrodes at a considerable distance from the stimulation site(s) (≥400 μm, Figs. 1A, 5A). Therefore, we were able to evoke fEPSPs that were sufficiently large and at the same time could be reliably potentiated in a pathway-specific manner. In our opinion, precise information about relative positions of recording and stimulating electrodes in a hippocampal slice is important for comparison of results between different laboratories. Having analysed 89 papers from a mouse knock-out plasticity phenotype database (A. Howell, M. Marshall, S. Grant, manuscript in preparation) which studied LTP of fEPSPs in the CA1 area, we were surprised to find that only ten of them mentioned the distance between stimulating and recording electrodes. Relative location of the recording electrode with respect to part of SR (proximal, medial or distal) was stated explicitly only in one of these 89 reports. It has been recognised however that relative dendritic location of electrodes could affect the degree of LTP in the hippocampus [29,50,56] and neocortex [57]. Therefore, we sought to investigate whether spatial differences in LTP existed in our preparation.

Using fairly dense 8 × 8 3D MEAs with inter-electrode distance of 100 μm we compared responses from three recording electrodes in the proximal, medial and distal parts of the along apical dendritic area of CA1 pyramidal cells (Fig. 1A). Applying baseline stimulation and subsequently TBS through one of the electrodes situated in the mid-distal part of SR on the border between CA1 and CA3 areas, we demonstrated that the largest potentiation of fEPSPs occurred in proximal dendritic sites within 50–80 μm of SP (Figs. 3, 4). This difference in potentiation could not be accounted for just by the fact that for a given submaximal stimulus, medial fEPSPs were larger that proximal ones (Fig. 2) and therefore less prone to potentiation. As follows from Fig. 4B, the ratio of post- to pre-TBS fEPSP was noticeably bigger in proximal sites. Another explanation for larger LTP at proximal part of SR could be contamination of proximal recording waveforms by population spikes that originated in the cell body. It should be noted that population spikes were not seen consistently in all recordings (for an example, see Additional file 1) and if observed, they were mostly pronounced at high stimulus strengths (Fig. 1B, electrode 5). Fig. 3A demonstrates that larger enhancement of proximal fEPSP (electrode 4) was recorded in the absence of a strong population spike (electrode 5) able to invade apical dendrites. Nonetheless, since amplitudes of population spikes are known to undergo much stronger potentiation compared to fEPSPs (so-called "E-S potentiation") [6,58-60], the fact that we measured amplitude rather than initial slope of our responses could lead to artificial overestimation of proximal LTP due to significant population spike contribution to compound fEPSP. We did not attempt measuring fEPSP slopes routinely, because our analysis software could calculate slopes only between integer milliseconds making it difficult to assign similar regions of the rising part of fEPSP among different recordings. In order to test if conventional slope calculation would yield consistently smaller estimates of LTP compared to peak amplitude measurements, we calculated slopes within initial one or two milliseconds of fEPSP traces for experiments depicted on Fig. 6A. No significant differences were observed in estimates of LTP when using slope or peak amplitude measurements in either 129S5 (peak: 191 ± 39 %; slope: 220 ± 57 %; *t*_(11)_=-1.81, *P *= 0.097) or C57 mice (peak: 187 ± 44%; slope: 200 ± 69 %; *t*_(13) _= -1.03; *P *= 0.323). Similarly, no large discrepancies between these two measures of LTP have been found in other reports [61,62].

Greater LTP in proximal dendrites could be a consequence of an altered balance between excitation and inhibition following LTP induction. Simultaneously with LTP of fEPSPs, tetanic stimulation can cause long-term depression of inhibitory signals [63]. On the other hand, since the density of inhibitory synapses is maximal in the proximal part of CA1 apical dendrites [64], then disinhibition could potentially have a greater effect on fEPSPs in this region.

Larger LTP at proximal as compared to distal SR recording sites was reported in conditions when pressure ejection of tetrodotoxin in SO was used to prevent post-synaptic spiking [56]. There are several methodological differences between the latter study and our experiments: we used mouse whole hippocampal slices and no pharmacological inhibitors, whereas Kolta *et al*. employed rat mini-slices of CA1 area and blockers of GABA_A _and GABA_B _receptors [56]. Nonetheless, collectively these data indicate that somatic population spikes are unlikely to be principal contributors to increased proximal fEPSPs after LTP induction. The cause of differences in LTP expression across SR levels may stem from a greater propensity of proximal dendrites to generate dendritic spikes. Current-source density analyses demonstrated that in response to the orthodromic stimulation CA1 proximal apical dendrites produce compound fEPSPs that contain a tetrodotoxin-sensitive component that precedes the population spike and is spatially distinct from synaptic currents [49,52,54]. It has been suggested that *in vivo *somatic action potentials are, in fact, initiated in proximal SR [53]. Dendritic spikes were also shown to be prerequisites of somatic firing, because even maximal stimulation of Schäffer collaterals failed to fire pyramidal cell bodies if tetrodotoxin was ejected locally onto proximal apical dendrites [65]. Of particular relevance to our data is the observation that induction of LTP leads to preferential enhancement of the proximal dendritic current sink [50]. Taken together these studies demonstrate that voltage-dependent conductances complement apical synaptic currents in shaping fEPSPs and amplify depolarisation and calcium entry during tetanic trains [66-68]. There is evidence about non-uniform distribution of voltage-dependent channels in CA1 apical dendrites [69], however, it is not quite clear which of them could account for the spatial gradient of LTP observed in this and other studies [29,56].

Two-pathway stimulation experiments (Fig. 5) have confirmed that increase of proximal fEPSPs, as a result of LTP, was specific to the tetanised pathway and did not reflect a generalised heterosynaptic enhancement of post-synaptic excitability. HFS and TBS markedly augmented fEPSPs that stayed elevated for at least an hour (Figs. 6A, 7A). The degree of LTP was comparable with estimates in other reports that employed similar protocols in mouse hippocampal slices [19,70-74]. Plasticity induced by HFS and TBS was entirely dependent on NMDA receptors, as incubation with a competitive NMDA receptor blocker completely abolished LTP (Figs, 6B, 7B). This is an agreement with published data on high sensitivity of LTP induced by these protocols to inhibition of NMDA receptors [71,75,76].

Brief episodes of TPS are known to evoke LTP which is dependent on activity of protein kinase A and mitogen-activated protein kinase ERK [42-44,71,77]. We were particularly interested in this form of plasticity as it strongly correlated with the learning ability of rats in the Morris water maze [46]. Also, impairment of TPS-evoked LTP that paralleled behavioural abnormalities was found in mutant mice lacking some intracellular signalling proteins [19,78,79]. Our initial attempts to induce LTP by 150 baseline strength pulses repeated at 5 Hz however were not successful. Most probably, this was because monopolar stimulation through MEA electrodes was not strong and co-operative enough at this frequency to elicit the complex spiking required for induction of LTP [44]. Having elevated stimulus strength to 150% of the baseline during TPS episode, we managed to evoke moderate but persistent potentiation in most of the slices (Fig. 8A; Additional file 2). Some reports mentioned the need for somewhat larger stimulus strengths to generate reliable complex spikes and stable LTP by TPS [79,80]. The relatively small degree of LTP observed by us might be explained also by the difference in incubation conditions. Most previous accounts of TPS LTP were based on hippocampal slices that were kept in interface chambers [43,44,46,71] which could impart different metabolic properties on brain tissue compared to submerged chambers [81]. Blocking NMDA receptors with 50 μM *D,L*-AP5 inhibited TPS-induced LTP (Fig. 8B) which agrees with published data [43,44,46]. Interestingly however, incubation with *D,L*-AP5 did not always abolish complex spiking in our slices (data not shown), as reported by others [46]. In the presence of *D,L*-AP5, LTP of fEPSPs was small but significant 110 ± 9% (t_(5) _= -3.32; *P *< 0.05). As overall TPS-induced LTP was quite modest in our experiments, the size of this previously documented NMDA-independent component [43,44,46] was minimal.

For our study, we intentionally chose C57 and 129S5 inbred mouse strains that are often used in the creation of genetically modified mice [82]. Several studies have pinpointed differences in synaptic plasticity, neurochemistry and behavioural performance among various inbred strains [83-87]. This variability could complicate comparative phenotyping of transgenic mice obtained on different backgrounds. For example, Nguyen and colleagues reported that TBS and multiple trains of HFS evoke lower levels of LTP in the CA1 area of 129/SvEms mice as compared to C57 strain [84,85]. In our experimental setting, we observed no difference in any kind of LTP between C57 and 129S5 strains (Figs. 6A, 7A, 8A). This outcome may seem somewhat surprising as 129S5 strain is closely related to 129/SvEms [88]. This discrepancy could be explained by methodological variations, in particular with regard to TBS that comprised twice as many stimuli in our experiments compared to reports [84,85]. On the other hand, Nguyen et al. detected differences in LTP between 129/SvEms and C57 strains after both one [84] and four [85] 1 s-long trains of 100 Hz, whereas we saw similar LTP levels in C57 and 129S5 mice after two such trains (Fig. 6A). Thus, it is hard to conclude whether discrepancy between our results and findings of Nguyen et al. are due to methodological differences or variations within 129 sub-strains [88]. Despite our experiments failed to reveal differences in LTP among C57 and 129S5 mice, they do not rule out the notion that inbred mouse strains are heterogeneous with respect to synaptic plasticity [83-87]. Clearly, more data from other strains are necessary to determine the extent of variation of plasticity properties in MEA experimental settings. Another ultimate test of the sensitivity of MEA-based approach to study LTP would be its ability to provide a clear differentiation between wild-type and mutant mice with well established plasticity phenotypes. Therefore, in our future experiments, we shall use several previously characterised knock-out mice to further validate the utility of the MEA platform for large scale plasticity screening. In summary, our study has shown that MEAs could be used to acquire large signals, comparable with fEPSPs recorded by standard extracellular recording techniques. Utilising MEAs, we were able to reliably induce large and persistent LTP of fEPSPs in hippocampal slices by established stimulation protocols. Consequently, MEAs should provide a powerful methodology for plasticity studies with the capacity to simultaneously collect information from several sites. The versatility of MEA-based approach in stimulation of the slice tissue by multiple electrodes makes it especially useful for synaptic tagging and spike-timing dependent plasticity experiments.

## Conclusion

Application of 3D MEAs allowed for a detailed examination of the synaptic transmission in acute slices of the mouse hippocampus. Simultaneous recording by several electrodes demonstrated a spatial gradient of LTP in SR of the CA1 area of the hippocampus and highlighted proximal regions of apical dendrites as sites where post-synaptic responses undergo largest plastic changes. LTP evoked by diverse stimulation patterns using MEAs was pathway-specific and dependent on the activity of NMDA receptors. Robust performance of 3D MEAs combined with the multiplexing potential of MEA technology proves that MEAs could be effectively used for phenotypic characterisation of transgenic animals.

## Methods

### Preparation of hippocampal slices

Experiments were performed on hippocampal slices obtained from 2–4 months old 129S5/SvEvBrd and albino C57BL/6J-*Tyr*^C-Brd ^mice bred at the Wellcome Trust Sanger Institute. Animals were killed by cervical dislocation in accordance with Schedule 1 to the U.K. Animals (Scientific Procedures) Act 1986. Whole brain was immediately transferred to a beaker containing ice-cold "cutting" solution of the following composition (in mM): sucrose 110, NaCl 60, NaHCO_3 _28, NaH_2_PO_4 _1.25, KCl 3, MgSO_4 _7, CaCl_2 _0.5, glucose 5, sodium ascorbate 0.6, phenol red 0.015. Prior to use, this solution was thoroughly saturated with a gas mixture of 95%O_2_/5%CO_2 _to maintain pH level within physiological range (7.25–7.35). Brain was allowed to chill for 2–3 min and then it was trimmed and mounted on the stage of a Vibroslice MA752 (Campden Instruments, Loughborough, UK) with SuperGlue in such a way, so that the blade would cut through hemispheres at an angle of 20–30° their horizontal planes [89]. "Cutting" solution in the temperature-controlled Peltier bath was cooled to 3.5–4.0°C and constantly bubbled with a mixture of 95% O_2 _and 5% CO_2 _. Slices were cut at 350 μm by stainless steel blades using minimal speed of blade advancement and vibrating frequency set at "6" mark. Eight to ten slices containing medial segments of the hippocampus with overlaying cortical areas were trimmed of the remaining tissue, placed into a well of a slice chamber (Fine Science Tools, Foster City, CA) and kept submerged under the constant flow (2 ml/min) of fresh artificial cerebrospinal fluid (ACSF) containing (in mM): NaCl 124, NaHCO_3 _25, NaH_2_PO_4 _1, KCl 4.4, MgSO_4 _1.2, CaCl_2 _2, glucose 10, phenol red 0.015. When slices were being transferred to the slice chamber, ACSF temperature was maintained at 23°C and then gradually increased to 29–31°C for the rest of the incubation period. Slices rested in these conditions for at least two hours before experiments commenced.

### Electrophysiological recording

The MEA60 electrophysiological suite was used for fEPSP recordings (Multi Channel Systems, Reutlingen, FRG). Two set-ups consisting of a MEA1060-BC pre-amplifier and a filter amplifier (gain 1100× or 550×) were run in parallel by a data acquisition card governed by MC_Rack 3.2.1.0 software. Raw electrode data were digitised at 10 kHz and recorded on a PC hard disk for further analysis. To record fEPSPs, hippocampal slices were placed into the well of an MEA biochip filled with ACSF. Two types of single-well biochips with 3D, tip-shaped electrodes were used: 1) 8 × 8 3D MEAs that had electrode height of 25–35 μm and electrode spacing of 100 μm; 2) 5 × 13 3D MEAs with electrode height of 35–45 μm and electrode spacing of 140 and 200 μm (Ayanda Biosystems, Lausanne, Switzerland). The bath was grounded via an Ag/AgCl pellet attached to the MEA amplifier ground socket. Slice position and contact with electrodes were secured by a nylon mesh glued to a flattened piece of platinum wire. In some experiments where exact positioning was vital, slices were immobilised by a silver ring with attached nylon mesh that was lowered vertically by a one-dimensional U-1C micromanipulator (You Ltd, Tokyo, Japan). MEA biochips were fitted into the pre-amplifier case and fresh ACSF was delivered to the MEA well through a temperature-controlled perfusion cannula that warmed perfusing media to 32°C. Monopolar stimulation of Schäffer collateral/commissural fibres through array electrodes was performed by STG2008 stimulus generator (Multi Channel Systems, Reutlingen, FRG). Biphasic (positive/negative, 100 μs/a phase) voltage pulses were used. Amplitude, duration and frequency of stimulation were controlled by MC_Stimulus II software. In one-pathway experiments utilising 8 × 8 3D MEAs, one electrode in the medial or distal part of SR near CA1/CA3 border was chosen for stimulation. A column of recording electrodes that was perpendicular to pyramidal cell layer was selected in the CA1 area. The shortest normal between this column and stimulation electrode was 400 or 500 μm. In two-pathway experiments conducted on 5 × 13 3D MEAs, a single principal recording electrode was picked in proximal part of CA1 SR. To stimulate control and test pathways, two stimulation electrodes were assigned on the subicular side and on the CA3 side of SR respectively. The distance from the recording electrode to the test stimulation electrode was 400–560 μm and to the control stimulation electrode 310–560 μm. To evoke orthodromic fEPSPs, stimulation electrodes were activated at a frequency of 0.02 Hz. Maximum possible stimulation of 3.7 and 4.5 V could be applied to electrodes using 8 × 8 and 5 × 13 3D MEAs respectively. Population fEPSPs of 1–4 mV amplitude were recorded in the CA1 area depending on the electrode location. In contrast to most recordings obtained by conventional glass electrodes, initially negative-going fEPSPs recorded by MEAs always had a pronounced positive shoulder throughout SR. We have not investigated the source of this phenomenon in detail, but as the waveform pattern was exactly the opposite in SO, (i.e. initially positive deflection and then a negative wave in response to stimulation of Schäffer collateral/commissural fibres) we reasoned that this secondary potential had a synaptic origin and was not an abnormal stimulation artefact. Following at least 15–30 min of equilibration period inside an MEA well, I/O relationships were obtained and baseline stimulation strength was set to evoke a response that corresponded to 40% of the maximal attainable fEPSP at the recording electrode located in proximal SR. Slices that had maximum responses less than 1 mV in the test pathway were discarded. LTP was induced after 30 min of stable baseline responses by applying patterned trains of stimuli to a single stimulation electrode in one-pathway experiments or to the test electrode in two-pathway trials. Three stimulation paradigms were used: 1) TBS that comprised three trains administered at 20 s intervals with 10 bursts given at 5 Hz per train and 4 pulses given at 100 Hz per burst. When working with 5 × 13 3D MEAs, baseline stimulation intensity was not altered during TBS, however in experiments that used 8 × 8 3D MEAs, it was increased to 1.25× baseline strength; 2) HFS that consisted of two trains administered at a 50 s interval with 100 baseline intensity pulses given at 100 Hz per train; 3) TPS that involved a single train of 150 pulses elicited at 5 Hz using 1.5× baseline stimulus intensity.

### Data analysis

Amplitude of the negative part of fEPSPs was used as a measure of the synaptic strength. Differences between groups were detected using mixed or repeated-measures ANOVA as appropriate. Bonferroni/Dunn's *post hoc *tests were done when the ANOVA *F*-value indicated significant differences (*α *= 0.05). LTP plots were normalised to the average of the first five baseline points. Statistical comparisons of normalised fEPSP amplitudes before and after LTP were performed by paired Student's *t*-test using averages of five data points just before and 60 min after LTP induction. Unpaired Student's *t*-test was used to examine differences in LTP levels between strains and also between *D*,*L*-AP5-treated and untreated slices. Values were considered to be different if *P *< 0.05. All reported values represented mean ± standard deviation with *n *indicating number of slices. Statistics were calculated using Statview 5.0.1 for Mac (SAS Institute, Cary, North Carolina) and Origin 7.0 (OriginLab, Northampton, Massachusetts).

## Abbreviations

129S5: 129S5/SvEvBrd mouse strain

C57: C57BL/6J-TyrC-Brd mouse strain

fEPSP: field excitatory post-synaptic potential

HFS: high frequency stimulation

LTP: long-term potentiation

MEA: multi-electrode array

NMDA: N-methyl-D-aspartate

SLM: stratum lacunosum moleculare

SO: stratum oriens

SP: stratum pyramidale

SR: stratum radiatum

TBS: theta-burst stimulation

TPS: theta-pulse stimulation

## Authors' contributions

MVK performed all the experiments described in Figures 1–6 and most of the experiments illustrated in Figures 7 and 8. NOA carried out some experiments shown in Figures 7 and 8, participated in the analysis of fEPSP data, performed data mining in the Plasticity Database and assisted in the slice preparation. SGNG co-ordinated experiments and contributed to the data analysis and interpretation. The study was conceived by SGNG and MVK. All authors have read and approved the final manuscript.

## Supplementary Material

Additional file 1 **An example of a laminar fEPSP profile in a hippocampal slice where no population spikes were elicited using maximal stimulation through an MEA electrode**. **A) **A 350 μm thick hippocampal slice was held over 8 × 8 3D MEA and a stimulation electrode (red circle) was chosen to excite Schäffer collateral/commissural fibres. A column of electrodes (grey circles) aligned in parallel with the direction of apical dendrites of CA1 pyramidal neurones was chosen to record synaptic responses in SLM (electrode 1), SR (electrodes 2–4) and SP/SO (electrode 5). Layers of cell bodies are delineated by the yellow dashed line. Distance between electrodes is 100 μm. Recordings from this slice are also presented in Fig. 3A. **B) **Waveforms of fEPSPs recorded by electrodes 1–5 in response to a maximal voltage step (biphasic positive/negative 3.5 V pulse, 100 μs/phase) applied to stimulation electrode. Note that on the border of SP and SO only positive field potential was recorded without a population spike. Click here for file

Additional file 2 **Sample recording that demonstrates pronounced complex spiking and robust LTP following TPS**. A) Alternating stimulation of control and test pathways was performed at baseline stimulus strength that elicited 40% of the maximum fEPSP (left column, black traces). Following 30 min of baseline recording, TPS of the test pathway was performed by 150 stimuli repeated at 5 Hz and the stimulation strength was increased to 1.5× of the baseline level. Blue traces represent waveforms of the 1^st^, 25^th^, 50^th^, 100^th ^and 150^th ^fEPSP in the TPS series. Note, that complex spiking achieves its maximum at approximately 50^th ^pulse and then amplitudes of the primary and secondary fEPSPs decrease. Right column illustrates fEPSP recordings 60 min after LTP induction (red traces) with overlaid corresponding baseline fEPSPs (black traces). As a result of TPS, fEPSP amplitude was enhanced in the test but not the control pathway. B) Plot of fractional fEPSP amplitudes in the control and test pathways before and after LTP induction. Amplitudes of fEPSPs during TPS episode, when stimulus strength was increased, are marked in blue colour. Note a transient depression of fEPSPs in both pathways after TPS. Click here for file
