# Supplementary figures and images for: Recording long-term potentiation of synaptic transmission by three-dimensional multi-electrode arrays
Source: BMC Neurosci. 2006 Aug 30;7:61. doi: 10.1186/1471-2202-7-61 (PMC1574331; doi:10.1186/1471-2202-7-61)

**A**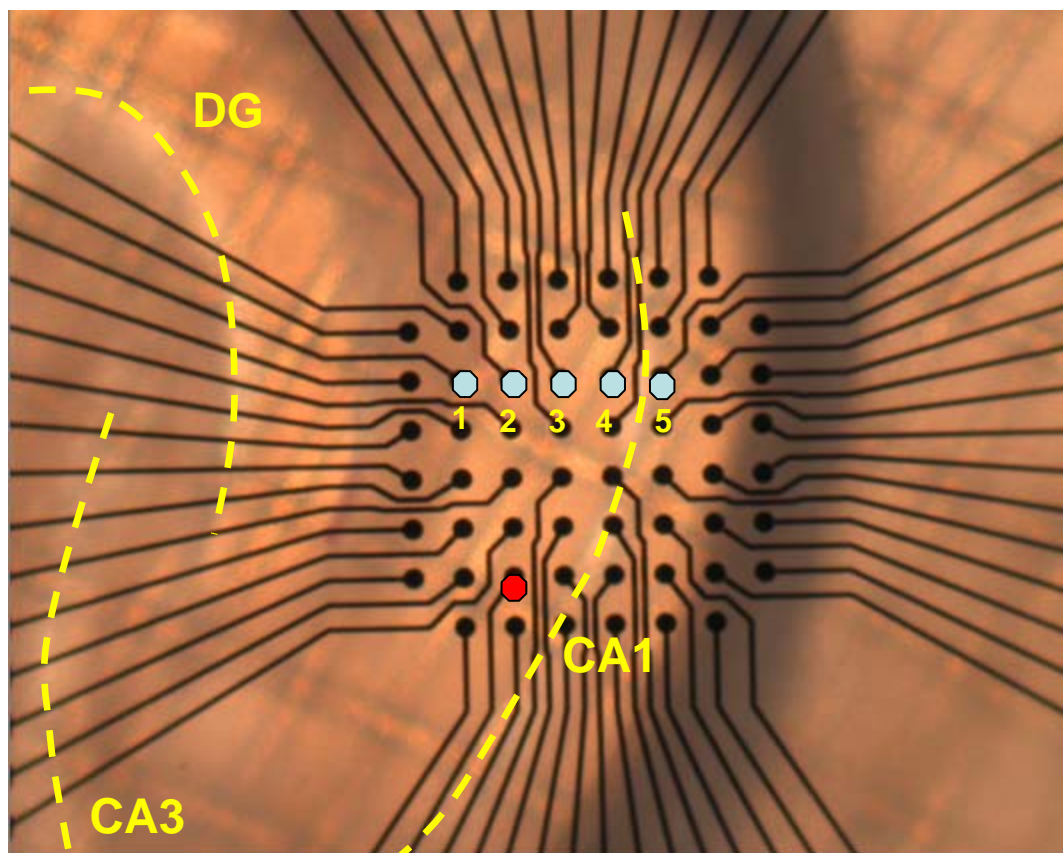**B**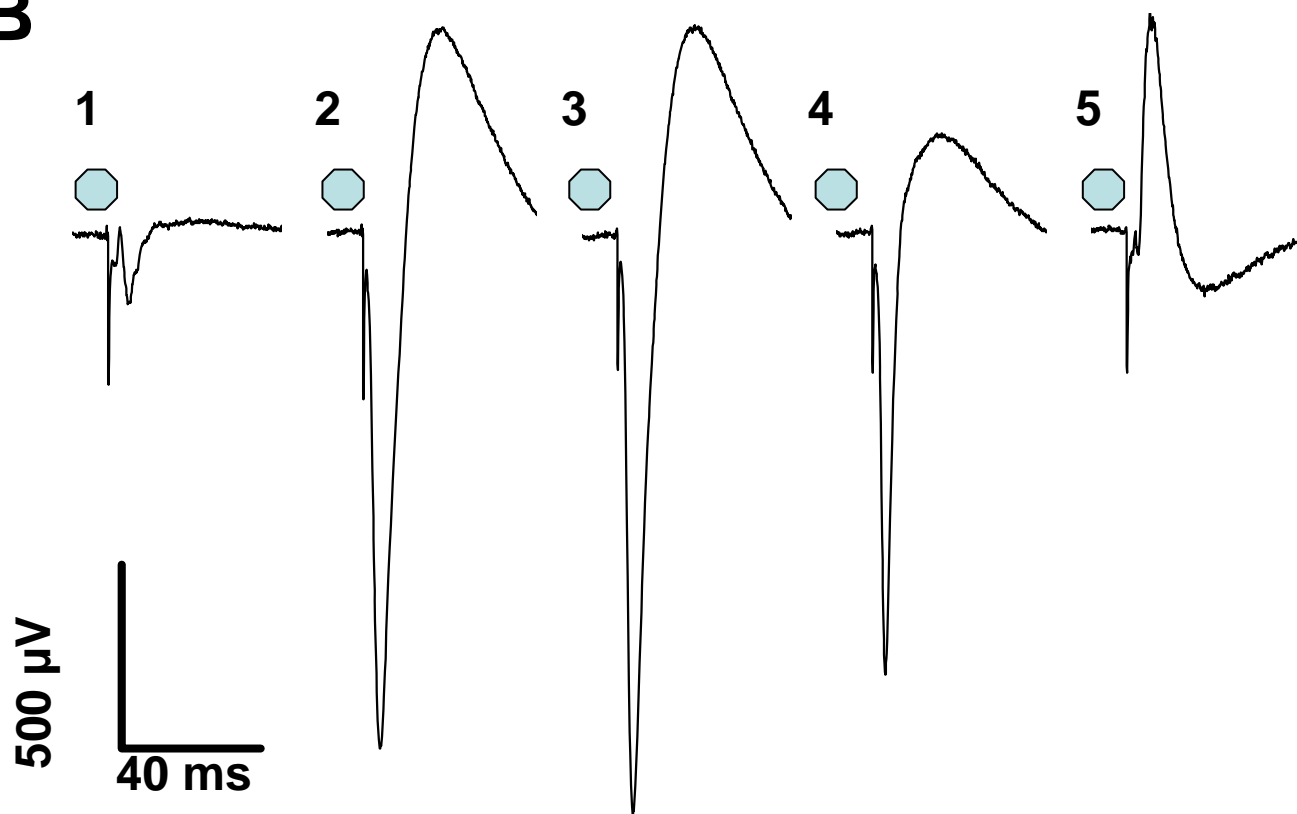

Supplement: Additional file 1 — An example of a laminar fEPSP profile in a hippocampal slice where no population spikes were elicited using maximal stimulation through an MEA electrode. A) A 350 μm thick hippocampal slice was held over 8 × 8 3D MEA and a stimulation electrode (red circle) was chosen to excite Schäffer collateral/commissural fibres. A column of electrodes (grey circles) aligned in parallel with the direction of apical dendrites of CA1 pyramidal neurones was chosen to record synaptic responses in SLM (electrode 1), SR (electrodes 2–4) and SP/SO (electrode 5). Layers of cell bodies are delineated by the yellow dashed line. Distance between electrodes is 100 μm. Recordings from this slice are also presented in Fig. 3A. B) Waveforms of fEPSPs recorded by electrodes 1–5 in response to a maximal voltage step (biphasic positive/negative 3.5 V pulse, 100 μs/phase) applied to stimulation electrode. Note that on the border of SP and SO only positive field potential was recorded without a population spike. [file 1471-2202-7-61-S1.pdf]

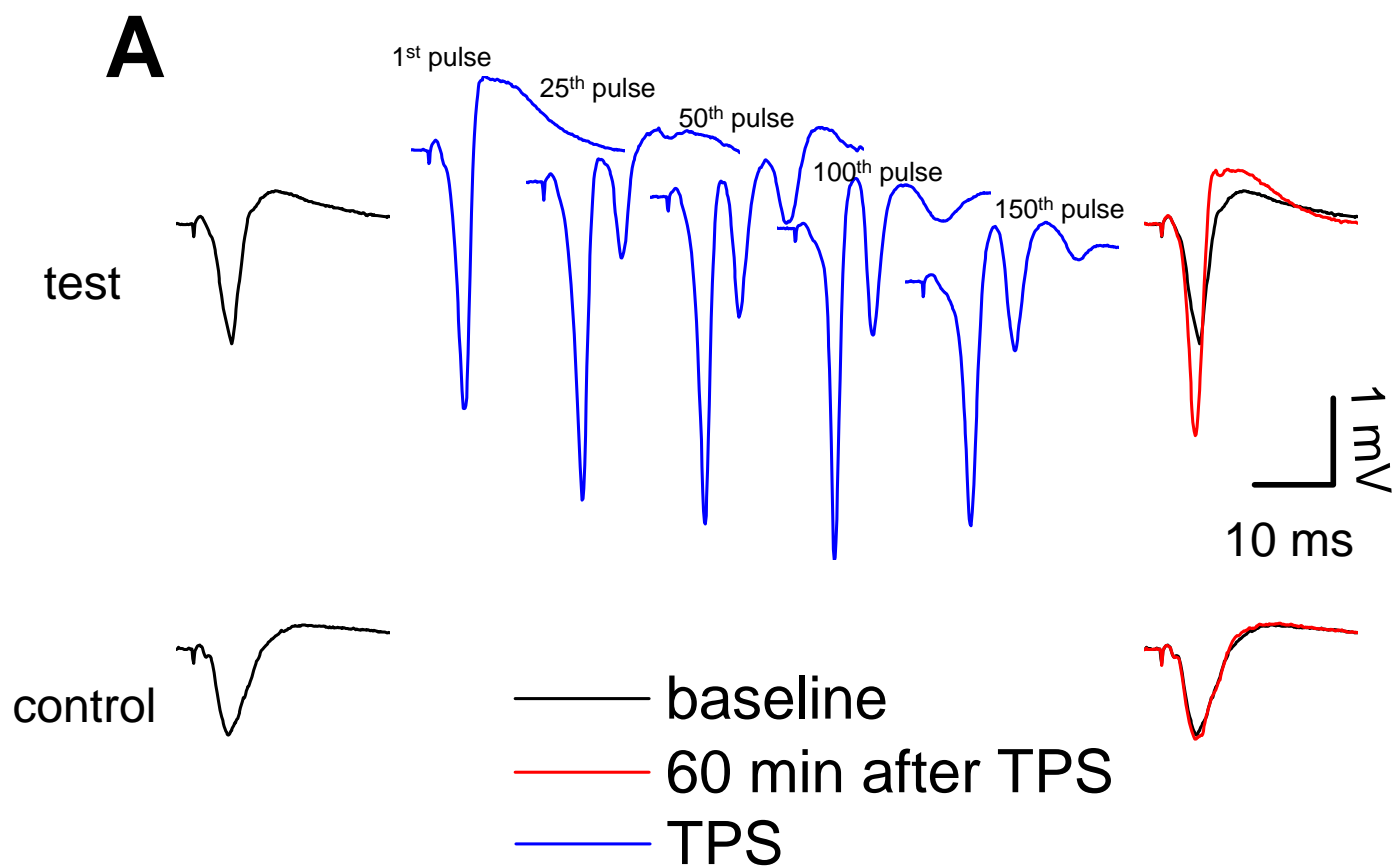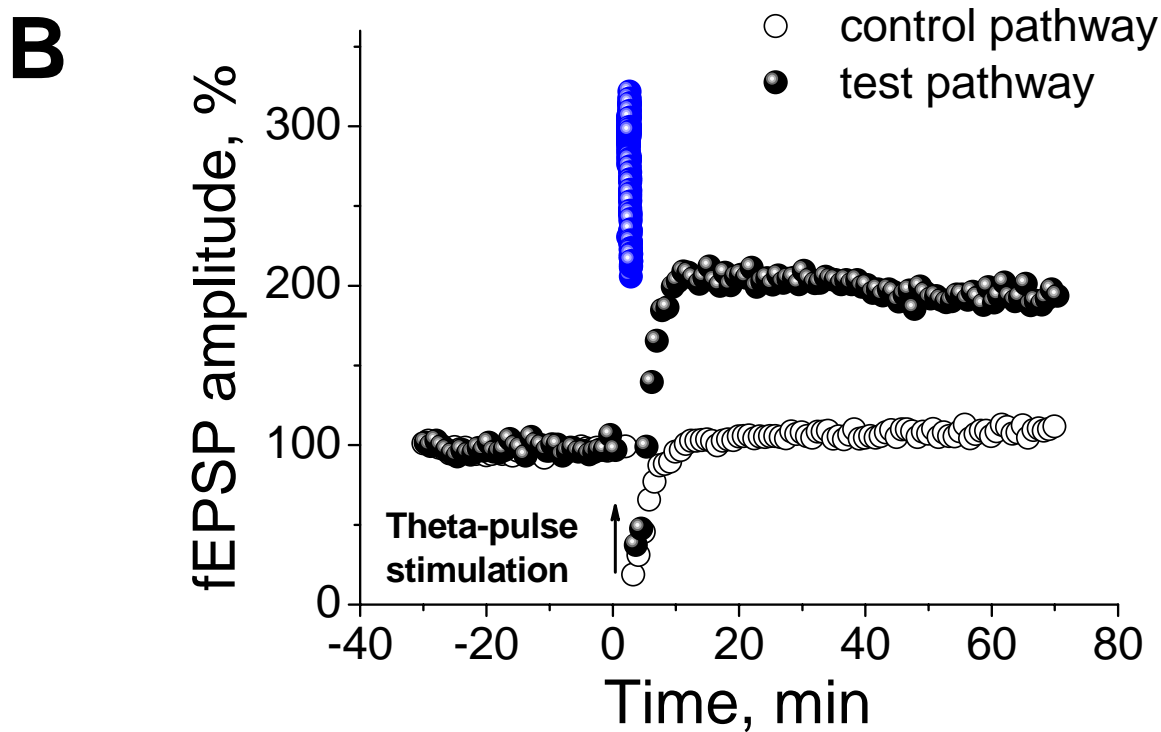

Supplement: Additional file 2 — Sample recording that demonstrates pronounced complex spiking and robust LTP following TPS. A) Alternating stimulation of control and test pathways was performed at baseline stimulus strength that elicited 40% of the maximum fEPSP (left column, black traces). Following 30 min of baseline recording, TPS of the test pathway was performed by 150 stimuli repeated at 5 Hz and the stimulation strength was increased to 1.5× of the baseline level. Blue traces represent waveforms of the 1st, 25th, 50th, 100th and 150th fEPSP in the TPS series. Note, that complex spiking achieves its maximum at approximately 50th pulse and then amplitudes of the primary and secondary fEPSPs decrease. Right column illustrates fEPSP recordings 60 min after LTP induction (red traces) with overlaid corresponding baseline fEPSPs (black traces). As a result of TPS, fEPSP amplitude was enhanced in the test but not the control pathway. B) Plot of fractional fEPSP amplitudes in the control and test pathways before and after LTP induction. Amplitudes of fEPSPs during TPS episode, when stimulus strength was increased, are marked in blue colour. Note a transient depression of fEPSPs in both pathways after TPS. [file 1471-2202-7-61-S2.pdf]
